# Functional Gastrointestinal Disease in Autism Spectrum Disorder: A Retrospective Descriptive Study in a Clinical Sample

**DOI:** 10.3389/fpsyt.2019.00179

**Published:** 2019-04-10

**Authors:** María José Penzol, Gonzalo Salazar de Pablo, Cloe Llorente, Carmen Moreno, Patricia Hernández, Maria Luisa Dorado, Mara Parellada

**Affiliations:** ^1^Child and Adolescent Psychiatry Department, Hospital General Universitario Gregorio Marañón, School of Medicine, Universidad Complutense, IiSGM, CIBERSAM, Madrid, Spain; ^2^Molecular Medicine Ph.D Program, University of Santiago de Compostela, Santiago de Compostela, Spain

**Keywords:** functional gastrointestinal disease, autism spectrum disorder, clinical comorbidity, prevalence, retrospective study

## Abstract

**Introduction:** Autism spectrum disorder (ASD) is a heterogeneous group of neurodevelopmental disorders with complex multifactorial etiologies. Medical comorbidities are common in ASD and include functional gastrointestinal disorders (fGID), which are reported in 30–70% of patients. In this research study, we aimed to systematically assess the prevalence of gastrointestinal problems in ASD and describe their clinical correlates.

**Methods:** In this retrospective study, we reviewed the medical records of all patients admitted to the Comprehensive Medical Program for ASD (AMITEA) at Gregorio Marañón University General Hospital from January 2012 to December 2015. All patients fulfilled the clinical criteria for ASD (DSM-IV-TR). In addition to fGID, epidemiological and clinical variables were collected at intake. Clinical and demographic features were compared among subjects with and without comorbid gastrointestinal problems.

**Results:** The analyses included all patients with documented information about presence/absence of fGID (*n* = 845; 95% of patients). Ages ranged from 1 to 53 years (mean = 10.52; SD = 8.92; 80.4% males). At least one fGID was present in 30.5% of patients, constipation being the most prevalent (47.4% of fGID patients); fGID were significantly associated with intellectual disability (ID) (*p* = 0.017), sleep disorders (*p* = 0.012), and prescription of psychopharmacological treatment (*p* = 0.019).

**Conclusions:** Almost one-third of ASD patients in our sample had at least one fGID. The presence of fGID was associated with ID, sleep problems and with behavioral problems (as measured by the prescription of psychotropic drugs). This subsample of ASD patients with fGID deserves particular attention in future research projects, focusing on specific phenotypic characteristics and overlapping biological markers that may underlie both pathologies.

## Introduction

Autism spectrum disorder (ASD) is a pervasive neurodevelopmental disorder characterized by impairments in social communication and restricted, repetitive patterns of behavior, interests, or activities (1). The neurobiological basis of ASD seems incontrovertible (2) even though the neurobiological mechanisms that result in the clinical phenotype remain to be fully elucidated. These include genetic factors, neuropathology, neurostructure, and brain networks (2). According to previous studies, more than 70% of individuals with ASD have other concurrent medical, developmental, or psychiatric conditions, which are frequently multiple (3–6).

It is widely reported that children with ASD are more likely to experience unmet medical needs compared with typically developing children (7). According to data from a US national survey, among children and adolescents with special health care needs, those with ASD are more likely to require specific health care services. This also holds true when compared with children who have special health care needs and behavioral, emotional, and developmental problems other than autism (8).

After years of debate about the presence of gastrointestinal (GI) symptoms in autism, there is currently a consensus to describe GI symptoms as a common comorbidity in patients with ASD even though the underlying mechanisms are largely unknown (9). A review published in Pediatrics reported that prevalence rates vary widely among studies and range from 9 to 91% in different samples, with great differences between retrospective and prospective studies (10, 11).

The GI problems, most commonly reported in autism, are chronic constipation, abdominal pain with or without diarrhea, and encopresis as a consequence of constipation (12). Furthermore, greater autism symptom severity is associated with increased odds of having GI problems (11, 13). Gastrointestinal symptoms are more likely to be reported by mothers in children with autism early in infancy than in children with typical development or developmental delay (14). Diagnosis of GI problems can be challenging in children with ASD because of their communication difficulties and complex behaviors (15), and therefore may be delayed (16), and problems frequently go undetected. GI symptoms in ASD may contribute to behavioral impairment, complicating clinical management. Gastrointestinal problems have been found to be associated with other behavioral and anxiety problems (17, 18).

Based on Roma Foundation working team, functional gastrointestinal disorders (fGIDs) are described as gut-brain interaction disorders, defined as a resulting on combination of symptoms affecting motility, hypersensitivity, immunity, and other alterations in mucose, causing an illness experience in patient's body; which are not caused by a anatomic or motility disorder (19).

A high prevalence of sleeping problems has been found in children with ASD (20–22), with longer sleep latencies and more difficulty going to bed and falling asleep (23). Co-occurrence of GI and sleep disturbances have been widely described in ASD, suggesting potential common pathophysiological pathways (24).

There are common pathophysiological mechanisms that account for both autism and epilepsy (25). ASD and epilepsy co-occur in approximately 30% of individuals with either condition (26). An epidemiological study detected an association between GID and epilepsy, identifying an increased risk for seizures co-occurring with GI symptoms in ASD patients (27).

It has been suggested that high medical comorbidity in ASD subjects may be due to common etiopathological factors or shared intermediate mechanisms, such as inflammation, immunity, redox status, etc. (16). However, an association of medical comorbidities with fGID in a sample of ASD subjects representative of the ASD general population has not yet been sufficiently explored.

Against this background, the purpose of this work was to explore fGID and associated conditions in a large sample of patients consecutively seen in a medical program for ASD subjects over a representative period of time.

In this study, we specifically evaluated (1) the prevalence of fGID in a large sample of individuals with ASD quasi-representative of the population of ASD patients in the Community of Madrid and (2) the sociodemographic and clinical characteristics and comorbidities in the subgroup of ASD patients with fGID.

## Materials and Methods

We conducted a retrospective study reviewing clinical records of all patients admitted to the Comprehensive Medical Program for ASD (AMITEA) (28) at Gregorio Marañón University General Hospital during the first four full years after implementing an electronic medical records system: from January 1, 2012 to December 31, 2015. The AMITEA program was founded in 2009 as a specialty care service for ASD patients with specific objectives: (1) to perform routine and symptom- or sign-driven medical assessments, (2) to facilitate access to the appropriate medical care using an individualized case management approach, (3) to centralize all possible medical and nursing procedures, and (4) to assess and treat comorbid psychiatric symptomatology (28). The only criteria for admission to the program are: (1) diagnosis of an ASD (all ages); (2) referral by a physician in the public sector; and (3) residing in the Community of Madrid (thus serving a population of 6,000,000). AMITEA is located in a tertiary hospital with all adult and pediatric specialties and more than 2,000 inpatient beds. Spain has a national health system that covers around 95% of the population. Given that the AMITEA program is open to all residents in the Community of Madrid Community with an ASD diagnosis (the only program of its kind) and admission is not restricted to individuals with health problems, the characteristics of program participants may be considered representative of the ASD general population in Madrid.

All participants fulfilled DSM-IV-TR criteria for ASD. Diagnoses were made by Child and Adolescent Psychiatrists, after a full psychiatric and developmental history, previous reports' review and observation of the child/adolescent. The Autistic Diagnostic Interview-Revised (ADI-R) and Autism Diagnostic Observation Schedule-Generic ADOS-G were used to support the diagnosis when clinicians deemed it necessary. When needed, ADI-R clinically trained and ADOS research trained child psychiatrists or psychologists did conduct these evaluations. All data was introduced in the electronic medical history of the Hospital at the time of intake, and electronically signed. In order to organize the information from the hospital medical history and report the data herein presented, we built a database in which patients' data were de-identified.

All data were collected as part of routine care, according to a protocol completed for every new participant registered in AMITEA. Sociodemographic and clinical variables were collected systematically by attending physicians (Child and Adolescent Psychiatrists). Data collected included age, sex, ASD diagnosis, type of ASD, and presence of fGID (gastrointestinal reflux, aerophagia, functional diarrhea, functional constipation, functional abdominal pain, cyclic vomiting). Specifics of the fGID, if present, were manually extracted from the electronic medical record and entered verbatim in the study database. Within variables deemed interesting with respect to gastrointestinal comorbidity, we only analyzed data that was included in the initial intake clinical history template. In this way, the data we report herein were available for all patients. GI information was categorized according to the domains in the ROME-III-R questionnaire for fGID (Appendix. Rome Foundation, 2006). Other comorbidities such as epilepsy, estimated intellectual disability (ID), and sleep disorders were also compiled. Behavioral and emotional problems were recorded as part of the psychiatric history, with no standard questionnaire; therefore, prescription of psychotropic treatment was gathered as a proxy for the behavioral problems (with clinical impact). Estimated ID was recorded as a dichotomous (yes/no) clinical variable after reviewing all the information in the medical record including psychometric tests, adaptive functioning questionnaires, and school placement and performance. The Institutional Review Board (IRB) of Gregorio Marañón University General Hospital approved the study.

## Analyses

We used descriptive statistics to characterize the study population including demographic variables and clinical characteristics. These were compared between patients groups with and without comorbid GI problems. A logistic regression model was then used to estimate odds ratios (ORs) and explore how factors differentially distributed between patients with and without fGID in the bivariate analyses contributed to the association with fGID. Therefore, we included age, sex, intellectual disability and sleep disorder as covariates in the multivariate analysis. Statistical analyses were performed with SPSS 21 for Windows software (IBM).

## Results

Our analyses included all patients with available information about presence/absence of fGID (*n* = 845; 95.6%) of a total sample of 879 persons admitted in the AMITEA program during 4 years; therefore, 34 patients were excluded from analysis since data of interest were not fully completed in clinical records. Ages ranged from 1 to 53 years, with a mean age of 10.52 (SD 8.92), and 19.6% were female. Children under 5 years of age comprised 25.2% of the sample (*n* = 213), 43% (*n* = 363) were ages 5–12, 15.9% (*n* = 134) were adolescents ages 13–17, and 16% (*n* = 135) were 18 or older. Four children aged 21–23 months were included in the program (following criteria as in the moderate-to-severe concern in Toddler module of the ADOS). Per DSM-IV criteria, all patients had a diagnosis of Pervasive Developmental Disorder; most patients (*n* = 476; 56.3%) had a diagnosis of Autistic Disorder, 210 (24.9%) had a diagnosis of Pervasive Developmental Disorder Not Otherwise Specified, 116 (13.7%) Asperger's Disorder, 2 patients (0.2%) had Rett's Disorder; and a small group (*n* = 41; 4.9%) did not had an specified subtype in the clinical record.

Thirty percent of subjects (*n* = 258, 30.5%) had an fGID, constipation being the most prevalent, occurring in almost half (*n* = 121; 47.6%) of the sub-sample with fGID; other symptoms identified were functional diarrhea (*n* = 29; 11.4%), gastrointestinal reflux (*n* = 20; 7.9%), functional abdominal pain (*n* = 16; 6.3%), aerophagia (*n* = 16; 6.3%), cyclic vomiting (*n* = 12; 4.7%), and other fGID not specifically reported (*n* = 40; 15.7%). Eighty-four patients (9.9%) of total sample were referred to a gastroenterologist for assessment, in this subgroup 75% presented fGID (χ^2^ = 87.609, *p* < 0.001).

With respect to the other comorbidities explored, 34.9% of the patients had a sleep disorder, 9.2% epilepsy, and 44.3% intellectual disability. More than half of the patients in the sample (55%) were prescribed psychopharmacological treatment. Psychotropic medication was more frequently used in patients over 12 years of age (χ^2^ = 79.184 *p* = 0.015), with a similar frequency in adolescents (79.9%) and adults (79.5%).

In contrast to the 30.5% of fGID in total sample, within the subsample of patients with ID, fGID was present in 34.8%, while in patients without ID, fGID was present in 65.2% (*p* = 0.017).

With respect to the differential sociodemographic and clinical characteristics of patients with and without fGID (see Table 1). The percentage of patients with ID was greater in the group with fGID (*p* = 0.017), as was in the sleep disorder group (*p* = 0.012), and the group with prescription of psychotropic drugs (*p* = 0.019). Other clinical variables did not differ in both groups. No differences concerning age and gender were found between groups with and without fGID.

**Table 1:** Sociodemographic and clinical characteristics in the groups with and without fGID.

	**fGID**	**No fGID**	**X^**2**^ test**	***p*-value**
	***N* = 258**	***N* = 587**		
Age groups			0.043	0.998
Age ≤ 5 (infants/toddlers)	24.8%	25.4%		
Age 5–12 (children)	43.4%	42.8%		
Age 13–18 (adolescents)	15.9%	15.8%		
Age ≥18 (adults)	15.9%	16.0%		
Sex (female)	20.9%	19.1%	0.389	0.573
ASD diagnostic category			3.634	0.603
Autistic Disorder	63%	57.5%		
Rett's Disorder	0%	0.4%		
Asperger's Disorder	12.2%	15.4%		
PDD NOS	24.8%	26.7%		
Known medical etiology	19.4%	18.5%	0.099	0.753
Epilepsy	11.8%	9.1%	1.457	0.247
Intellectual disability	51.2%	41.3%	5.946	0.017*
Sleep disorder	42.7%	33.2%	6.878	0.009*
Pharmacological treatment	65.3%	56.4 %	5.563	0.019*

*PDD-NOS, Pervasive Developmental Disorder-Not otherwise specified*.

* *Bold values indicate statistically significant difference (p < 0.05)*.

Most patients with sleep disorders (71.8%) were on psychopharmacological treatment, while only 28.2% of patients with sleep disorders were receiving no psychotropic medication (χ^2^ = 31.732, *p* < 0.001).

Exploring eventual predictors of fGID, we examined the variables associated with functional Gastrointestinal Disorders (see Table 2), logistic regression analysis showed that intellectual disability was significantly associated with fGID (OR = 1.490, 95% CI: 1.081–2.055; *p* = 0.015) and sleep disorder (OR = 1.502, 95% CI:1.107–1.2036; *p* = 0.009) were significantly associated with fGID. Result remained significant in the multivariate analysis when OR was adjusted for age, sex, and sleep problems; and also when OR for sleep problems when covariated by age, sex, and intellectual disability. Due to the high association between sleep disorder and psychopharmacological treatment, this last variable was not included in the regression.

**Table 2:** Functional Gastrointestinal Disorder.

	**Unadjusted OR**	**(95% CI)**	***p*-value**	**Adjusted OR^a/b^**	**(95% CI)**	***p*-value**
Age	1.002	0.986–1.019	0.801	–	–	–
Sex (male)	0.891	0.619–1.082	0.533	–	–	–
ASD diagnostic category	1	0.457–1.142	0.164	–	–	–
Autistic disorder	0.722		0.361	–	–	–
Asperger's Disorder	0.848	0.595–1.208		–	–	–
PPD NOS				–	–	–
Known medical etiology	0.942	0.649–1.367	0.753	–	–	–
Epilepsy	1.348	0.829–2.192	0.229	–	–	–
Intellectual disability	1.490	1.081–2.055	0.015*	1.402^a^	1.001–1.962	0.049*
Sleep disorder	1.502	1.107–2.036	0.009*	1.551^b^	1.112–2.164	0.010*
Pharmacological treatment	1.455	1.065–1.988	0.019*	–	–	–

a *Adjusting for age, sex, and sleep disorder*.

b *Adjusting for age, sex, and intellectual disability*.

*PDD-NOS, Pervasive Developmental Disorder-Not otherwise specified*.

* *Bold values indicate statistically significant difference (p < 0.05)*.

## Discussion

In this sample of 845 patients with ASD seen in a specialty care program for ASD patients in a tertiary hospital, almost a third had an fGID, constipation being the most prevalent (47%). The presence of fGID was associated with ID, sleep problems and with behavioral problems (as measured by the higher percentage of children in the ASD group with fGID treated with psychotropic drugs), but it was intellectual disability and sleep problems that stood out as the most significant associations with fGID once other variables were considered. No differences were detected in the prevalence of fGID among age groups or ASD diagnosis category.

In this large ASD sample, relatively representative of ASD subjects in a general population of around 6,000,000, there emerges a subgroup of patients (around 25% of the sample) characterized by GID and ID. This group of patients has concomitant associated health problems such as sleep and behavioral problems. Although parents with ID report fGID only a bit more frequently than parents of children without ID (34.8 vs. 30.5%, *p* = 0.017), the percentage could well be higher, taking into account the difficulties that ASD+ID patients may have communicating symptoms, especially if those include subjective perceptions such as pain or discomfort.

Our finding of nearly one-third of ASD patients presenting with fGID adds to the previous literature. A recent large population-based study (17) showed that 7.5% of ASD participants had a gastrointestinal problem, the most common being constipation. One-third of ASD patients presenting with fGID in a tertiary hospital is a higher figure than the 11.7% found in a recent study that automatically searched a large sample of medical records from four general hospitals (29) for comorbidities. Our data is likely more accurate, as the presence of fGID is systematically recorded at the intake visit in a program specifically designed to meet the medical needs of patients with ASD (2) referred by general practitioners. The nature of this program, designed to improve access to specialist care via a case manager, and the nature of the healthcare system in Spain, which is publicly funded and available to all citizens, results in a case load of patients either seen for any medical (including psychiatric) comorbidity or simply registered in the program should they need any specialty.

In our sample, functional gastrointestinal disorders were frequently associated with sleep disorders. According to the literature, GID and sleep problems are prevalent in pediatric samples without autism (around 45%) (30). In samples with autism, sleep problems are more prevalent than in general population; two-thirds of children with ASD have chronic insomnia (21), which may cause daytime behavior, memory, and learning problems in patients, and significant stress in caretakers (20). Medical conditions more frequently overlapped in autism samples; some authors have even suggested that patients with GI and/or sleep problems may represent a differential subtype of ASD with potential common pathophysiological pathways (22, 24, 27). GIDs have been described as a risk factor for sleep problems in children and adolescents with idiopathic autism (31).

Although the direction of the causal relationship between sleep problems and fGID is not known in many of the cases, early identification and treatment of GI disorders could improve sleep and help increase functionality and well-being in patients with ASD. In some cases, sleep problems clearly seem to arise from abdominal discomfort. As an example, a high percentage (40%) of some monogenic forms of autism, such as Phelan-McDermid syndrome, present a specific gastrointestinal disorder, namely gastroesophageal reflux (32). This is frequently associated with sleep problems. Understanding the origin and associations of sleep problems in specific cases may help put them in the appropriate place and determine the specific treatment intervention (in this case, antacid treatment), preventing children taking ineffective and potentially damaging treatments (e.g., psychotropics for sleep disturbances).

GI problems have been described in the literature in association with behavioral problems in patients with ASD (6, 22) and also with autism severity (17, 18, 33). Although our data focused only on the use of psychopharmacological treatment, it could be considered an approximation of the presence of this problem in our sample.

Identifying clinical phenotypes in ASD via medical comorbidities may be a promising approach for improving our understanding of the pathophysiology of subgroups of patients and optimizing therapeutic interventions (34). Wasilewska and Klukowsky suggest comorbidity of GI disorders and ASD as a new endophenotype and propose treating it as an “overlap syndrome” through different mechanisms. These mechanisms include multilevel pathways in the gut-brain axis contributing to alterations in behavior and cognition (16).

There is considerable evidence that the gut-brain axis is involved in the etiology of autism. This is based on a number of studies focusing on permeability of the intestinal mucosa, abnormal gut development, leaky gut, and others, with different mechanisms involving different systemic processes and the intermediate mechanism, many of those pointing toward a systemic chronic pro-inflammatory status. Furthermore, for the last several years, there has been an escalating number of studies showing changes of gut microbiota in patients with ASD (35).

## Limitations

We are aware that this study is subject to some limitations. Above all, it is a retrospective study, based on data recorded in medical records with only part of the clinical data potentially relevant to the topic of this study and systematically gathered. Since this is a transversal study, no data is available concerning how treatment of functional gastrointestinal symptoms could change patients' behavior. In addition, some clinical data potentially relevant for interpreting our results was not systematically recorded and could not therefore be analyzed.

Despite the above limitation, our study has important strengths. The major strength is the large sample size and its particularities, making it a sample quite representative of a population of children and adults with ASD.

In conclusion, a large sample of ASD patients from a tertiary hospital shows a subgroup of patients with fGID, half of them with intellectual disability and frequently with comorbid sleep problems and/or behavioral problems. This is a subgroup of patients with greater medical needs that need to be addressed, on top of their autistic core symptoms, in order to improve their well-being and consequently their adjustment.

## Ethics Statement

This study was conducted following the recommendations of the IRB of our institution and the Spanish Agency of Medicines and Medical Devices (AEMPS). Waiver of individual informed consent was approved by the IRB.

## Author Contributions

MJP and MP were responsible for conception and design of the study. MJP, GS, CL, PH, CM, MD, and MP were responsible for the clinical interview with patients and recorded data from clinical records. MJP and GS conducted the database generation and drafted the article. MJP and MP performed statistical analysis and data interpretation. MJP, GS, CL, PH, CM, MD, and MP reviewed the manuscript, made substantial contributions to conception and data acquisition and interpretation, making modifications as appropriate to the work in progress. All authors agree with the final content of this work.

### Conflict of Interest Statement

The authors declare that the research was conducted in the absence of any commercial or financial relationships that could be construed as a potential conflict of interest.
